# The analysis of relationship between selected sociodemografic factors and disorders of speech organs in Parkinson`s patients

**DOI:** 10.1186/s12883-017-1003-2

**Published:** 2017-12-21

**Authors:** Wioletta Pawlukowska, Karolina Skonieczna-Żydecka, Iwona Rotter, Krystyna Honczarenko, Przemysław Nowacki

**Affiliations:** 1 Department of Medical Rehabilitation, Chair and Clinic of Neurology, Pomeranian Medical University, Unii Lubelskiej 1, 71-252 Szczecin, Poland; 2 Department of Biochemistry and Human Nutrition, Pomeranian Medical University, Broniewskiego 21, 71-460 Szczecin, Poland; 3 Department of Medical Rehabilitation, Pomeranian Medical University, Żołnierska 54, 71-210 Szczecin, Poland; 4 Chair and Clinic of Neurology, Pomeranian Medical University, Unii Lubelskiej 1, 71-252 Szczecin, Poland

**Keywords:** Parkinson’s disease, Speech, Articulatory, Gender

## Abstract

**Background:**

Evaluation of speech disorders in PD taking into account sociodemographic conditions is not frequent. This paper aims to establish correlations between articulation disorders in PD patients and factors such as the patients’ sex, age, education and residence.

**Methods:**

The study included 92 patients with idiopathic PD diagnosed by means of multiple neurological examinations, biochemical tests, MRI and CT scanning carried out in accordance with the United Kingdom Parkinson’s Disease Society Brain Bank (UKPDSBB) criteria. A speech and language test involved the assessment of the mobility of the speech organs as well as the reflexes inside the oral cavity. Frenchay Dysarthria Assessment was applied for an objective evaluation of dysarthria.

**Results:**

The study revealed the existence of significant relationship between the functionality of articulators in PD patients and their education and residence. Big city dwellers demonstrated lower incidence of disorders within speech organs, particularly those affecting mobility of the soft palate while eating. Disorders of moderate intensity were more frequently found in subjects living in villages. Subjects with a university education displayed better position of the lips at rest and better performance of both lips and the mandible while speaking.

**Conclusions:**

Abnormal functioning of the articulatory organs was observed more frequently in PD patients residing in rural areas than in those inhabiting urban areas. As for education, our cohort university graduates displayed a better position of the lips at rest and better performance of the lips and jaw during speaking than those with secondary and vocational education.

## Background

Parkinson’s disease (PD) is one of the most frequently occurring neurodegenerative conditions of the central nervous system. In many cases its onset is heralded by gradually increasing speech decline. Speech disorders in PD are normally associated with the pathogenetic conditioning of the disease, particularly bradykinesia and rigidity. Speech and voice evaluation is usually carried out in the context of duration and severity of the disease as well as L-dopa dosage [1–3].

So far a limited number of studies evaluating speech disturbances in PD patients have been documented and published. Some researchers focused mainly on the abnormality of movement within the lips and jaw [4–6]. It was established that the slowness of the lower lip movement was related to dysarthric articulation disorders. Other researchers claimed that PD constitutes a factor contributing to the impairment in the mobility of the speech organs [7–10]. Reduced stability of movement coordination in the speech organs in PD patients was also shown [11].

Available publications describing speech disorders in PD patients in the context of demographic or social circumstances such as sex, age, education or place of residence, provide inconclusive results [4]. PD generally prevails in the elderly with the mean age of 58 years. Elderly people tend to be afflicted with speech disorders due to anatomic changes, loss of teeth and lower muscle tone [11, 12]. It has been established that PD patients lose the loudness of voice with age [13]. Other studies of voice loudness in PD patients indicate that there is a connection between patients’ age and pitch lowering [14]. The latest research into the performance of the articulatory organs carried out on elderly subjects and PD patients in an initial stage of the disease shows that there is no difference between those groups [15]. Studies into the relationship between sex and the severity of articulatory disorders in PD patients also provided ambiguous results.

Some researchers point to the existence of differences between the loudness of vowels uttered by male and female PD patients.

Several studies demonstrated that the incidence of PD in ethnic groups populating the same place is similar, but may vary depending on the geographical location of the place [16, 17]. It is assumed that living in the countryside contributes to a higher incidence of PD [18, 19]. This is associated with the impact of environmental factors. We attempted to find out whether those factors could also be responsible for speech deterioration in PD or an early onset of the articulatory organs disorders in PD patients.

A limited number of papers which would attempt to analyse the course of PD in patients of various educational backgrounds encouraged us to look into this unexplored question. Patients with a university education tend to make more effort to preserve their communication skills and overall bodily fitness. They are also less likely to display deterioration of cognitive functions [20, 21]. A better education normally means a higher economic status, better quality of life and a better access to healthcare [22]. Hence, it can be assumed that the quality of articulation in well-educated PD patients will be different from that in their less educated counterparts. Any publications on the relationship between the severity of articulation impairment in PD and the patients’ residence are also hard to come by.

Some researchers claim that articulation disorders in PD are caused by progression of the disease [23]. Further research into the issue seems to be well worthwhile as the ambiguities concerning the functioning of the articulatory organs and the risk factors responsible for their deterioration are numerous. Looking into the correlation between the incidence of the articulatory organs disorders and variables like sex, age, place of residence or education might help to understand the specifics of the symptoms better, give a more effective diagnosis and plan the rehabilitation of PD patients more effectively.

## Methods

The study was carried out on a cohort of 92 patients with diagnosed idiopathic PD who met the following criteria: age over 40, lack of significant psychotic changes, able stadium 1-4 on a scale M.M. Hoehn and M.D. Yahr (H&Y), no stupor symptoms present, retained L-dopa response, absence of clinical depression or mood disorders other than those relating to the ‘off’ phase. Criteria of exclusion included: undergone deep brain stimulation procedure (DBS), substantial discoordination of movement deficit, acute dyskinesias, pathological changes in the larynx, cognitive functions disorders (the Mini Mental State Examination scale was applied and subjects who scored ≤24 points were excluded from the cohort), other neurological diseases.

The study group contained 33 women (35.9%), and 59 men (64.1%), aged between 26 and 86 (mean age 65.2). 55 (59.8%) were big city dwellers, 24 (26.1%) lived in towns and 13 (14.1%) in villages. Seventeen patients had a university education, 28 (30.4%) completed secondary schools, 30 (32.6%) were trade school graduates and the remaining 17 (18.5%) received a primary education.

### Data collection

PD was diagnosed by means of multiple neurological examinations, biochemical tests, Magnetic resonance imaging (MRI) and Computed tomographic (CT) scanning in accordance with the United Kingdom Parkinson’s Disease Society Brain Bank (UKPDSBB) criteria. Duration of the illness, measured from the occurrence of the first symptoms of PD, varied from 1 month to 27 years (mean length 7.5 years). Further examination of the group using the Unified Parkinson’s Disease Rating Scale (UPDRS) produced scores ranging between 10 and 70 pts. (mean score 37.2 pts.). The study subjects had not received any neurological speech therapy. Daily intake of L-dopa ranged from 150 to 2000 mg (mean dosage 570.9 mg).

The data concerning gender, age, education and place of residence were collected by means of our own questionnaire.

Performance of the articulatory organs was assessed by means the standardized scale Frenchay Dysarthria Assessment (FDA). One of the most important tests for evaluating articulation organs is the FDA. The FDA is a standardized test which relies on a 9-point rating scale applied to a patient. It provides information based on the observation of the oral structures, functions and speech. It has good feasibility (missing data <5%), a high reliability of the total score (0.94), an excellent inter-rater agreement for the total score (0.96) and moderate to large construct validity for 81% of its items. It is well correlated with the gold standard for disease severity assessment in PD, the Movement Disorder Society -Unified Parkinson’s disease rating scale (MDS-UPDRS). The test evaluates the following functions: saliva control, swallowing, breathing, tongue movements, lips, the soft palate, the jaw, length of phonation as well as the pitch and loudness of voice. A 5-point rating scale (a – e) is used for the assessment, where letter ‘a’ represents norm, ‘b’ mild severity, ‘c’ moderate, ‘d’ considerable severity, ‘e’ profound severity. FDA is also used to assess the severity of the articulatory organs disorders and to monitor the effects of treatment [24]. The test was conducted by a clinical speech therapist with 12 years experience of treating neurological conditions, predominantly PD. We used an older version of the test because of the jaw test, which currently is not available in the updated version.

Patients with considerable deviation from the norm were referred for laryngological consultations so that any other conditions within the speech organs could be ruled out.

The subjects were examined in ‘OFF’ phase. Patients are considered to be ON when medication is working and OFF when the benefit abates. Some scales assess motor manifestations or complications of treatment, and ratings may vary tremendously depending upon whether observations are recorded during the ON or OFF phase for those with such medication-induced fluctuations [25].

The study was approved by the Pomeranian Medical University Commission – of Ethics -Resolution no KB-0012/07/10.

#### Statistical analysis

The distribution of continuous variable (age) was verified by the Shapiro-Wilk normality test. To verify whether speech and language test results were determined by selected socio-demographical indices χ^2^ (relation to gender, place of residence and level of education), Kruskall-Wallis or Mann-Whitney (in relation to age) tests were applicated, as appropriate. The statistical significance was adopted at two-side *P* value <0.05. All statistical analyses for this study were performed using the StatView computer software version 5.0 (SAS Institute Inc. Cary, NC, USA) [26, 27].

## Results

We successfully conducted speech and language evaluations in all patients (*n* = 92, 100%) recruited for the study. We found no association between the sex and age of the study participants and each determinant of the mobility of the speech organs as well as the reflexes inside the oral cavity. The results are presented in Tables 1 and 2 respectively.

**Table 1:** Study group characteristics

Variable	Mean	Standard Deviation
Age (years)	65,1	9,5
Duration of PD (years)	7,5	5,3
L-dopa dose (g)	570,9	404,6
UDPRS-III	17,2	7,2
UDPRS	37,2	16,5

**Table 2:** The association between age and particular speech determinants in the study group by means of Kruskall-Wallis test

Age vs:	Rating	Numbers	Mean rank	H	*p*
Cough	a	79	45.89	2.25	0.52
	b	10	52.50		
	c	2	56.25		
	d	1	14.50		
Swallowing	a	63	46.13	0.47	0.78
	b	26	48.42		
	c	3	37.66		
Drooling	a	31	46.79	0.32	0.85
	b	57	46.86		
	c	4	39.12		
Respiration at rest	a	72	46.78	1.45	0.48
	b	19	47.10		
	c	1	14.50		
Respiration in speech	a	52	47.08	1.62	0.65
	b	32	45.73		
	c	7	50.21		
	d	1	14.5		
Lips at rest	a	29	49.14	2.45	0.29
	b	61	46.16		
	c	2	18.75		
Lips spread	a	17	34.5	5.99	0.11
	b	51	47.18		
	c	18	50.64		
	d	6	62.25		
Lips sealed	a	43	46.24	2.58	0.46
	d	25	43.22		
	c	20	53.55		
	d	4	34.50		
Lips alternate	a	29	47.69	2.65	0.45
	b	54	45.06		
	c	8	55.94		
	d	1	14.5		
Lips in speech	a	28	49.09	3.75	0.29
	b	57	44.31		
	c	6	60.58		
	d	1	14.5		
Jaw at rest	a	73	47.31		0.57
	b	19	43.39		
Jaw in speech	a	43	43.08	2.74	0.25
	b	42	47.64		
	c	7	60.64		
Palate while eating	a	62	47.59	0.32	0.85
	b	28	44.19		
	c	2	45.00		
Palate maintenance	a	48	45.99	3.07	0.21
	b	40	49.27		
	c	4	24.87		
Palate in speech	a	47	46.17	0.26	0.88
	b	42	47.37		
	c	3	39.50		

Statistical analyses proved that the place of residence determines only the evaluation of the palate while eating (*p* = 0.017). A majority of big town dwellers (*n* = 43, 78%) demonstrated normal movement of the vocal organs. By contrast, a mild disorder of speech organs occurred in 61% of village residents. Detailed results are presented in Table 3. Furthermore, we found that a better education background of the patients corresponded with higher performance of the lips at rest, in motion and while speaking spontaneously as well as more efficient motion of the lower jaw in speech (0.0002; 0.0252; 0.0105; 0.0333 respectively) (Tables 4 and 5).

**Table 3:** The speech tests results in terms of gender calculated by χ^2^ analyses. Data are presented as number of cases

FDA indice	Rating	Gender				*p*
		Male (*n* = 59)	%	Female (*n* = 33)	%	
Cough	a	51	86.4	28	84.8	0.84
	b	6	10.2	4	12.1	
	c	1	1.7	1	3.0	
	d	1	1.7	0	0.0	
Swallowing	a	40	67.8	23	69.7	0.41
	b	16	27.1	10	30.3	
	c	3	5.1	0	0.0	
Drooling	a	18	30.5	13	39.4	0.25
	b	37	62.7	20	60.6	
	c	4	6.8	0	0.0	
Respiration at rest	a	45	76.3	27	81.8	0.67
	b	13	22.0	6	18.2	
	c	1	1.7	0	0.0	
Respiration in speech	a	37	62.7	15	45.5	0.27
	b	18	30.5	14	42.4	
	c	3	5.1	4	12.1	
	d	1	1.7	0	0.0	
Lips at rest	a	16	27.1	13	39.4	0.30
	b	41	69.5	20	60.6	
	c	2	3.4	0	0.0	
Lips spread	a	11	18.6	6	18.2	0.99
	b	33	55.9	18	54.5	
	c	11	18.6	7	21.2	
	d	4	6.8	2	6.1	
Lips sealed	a	32	54.2	11	33.3	0.06
	d	16	27.1	9	27.3	
	c	8	13.6	12	36.4	
	d	3	5.1	1	3.0	
Lips alternate	a	19	32.2	10	30.3	0.75
	b	33	55.9	21	63.6	
	c	6	10.2	2	6.1	
	d	1	1.7	0	0.0	
Lips in speech	a	18	30.5	10	30.3	0.89
	b	36	61.0	21	63.6	
	c	4	6.8	2	6.1	
	d	1	1.7	0	0.0	
Jaw at rest	a	46	78.0	27	81.8	0.66
	b	13	22.0	6	18.2	
Jaw in speech	a	29	49.2	14	42.4	0.12
	b	28	47.5	14	42.4	
	c	2	3.4	5	15.2	
Palate while eating	a	36	61.0	26	78.8	0.17
	b	21	35.6	7	21.2	
	c	2	3.4	0	0.0	
Palate maintenance	a	28	47.5	20	60.6	0.21
	b	27	45.8	13	39.4	
	c	4	6.8	0	0.0	
Palate in speech	a	27	45.8	20	60.6	0.22
	b	29	49.2	13	39.4	
	c	3	5.1	0	0.0

**Table 4:** The FDA indices in terms of place of residence by means of χ^2^ analyses Data are presented as number of cases

FDA indice	Rating	Place of residence						*p*
		Big city (*n* = 55)	%	Town (*n* = 24)	%	Village (*n* = 13)	%	
Cough	a	45	81.8	22	91.7	12	92.3	0.84
	b	7	12.7	2	8.3	1	7.7	
	c	2	3.6	0	0.0	0	0.0	
	d	1	1.8	0	0.0	0	0.0	
Swallowing	a	41	74.5	12	50.0	10	76.9	0.22
	b	12	21.8	11	45.8	3	23.1	
	c	2	3.6	1	4.2	0	0.0	
Drooling	a	20	36.4	7	29.2	4	30.8	0.85
	b	32	58.2	16	66.7	9	69.2	
	c	3	5.5	1	4.2	0	0.0	
Respiration at rest	a	43	78.2	17	70.8	12	92.3	0.54
	b	11	20.0	7	29.2	1	7.7	
	c	1	1.8	0	0.0	0	0.0	
Respiration in speech	a	30	54.5	12	50.0	10	76.9	0.69
	b	19	34.5	10	41.7	3	23.1	
	c	5	9.1	2	8.3	0	0.0	
	d	1	1.8	0	0.0	0	0.0	
Lips at rest	a	22	40.0	5	20.8	2	15.4	0.25
	b	32	58.2	18	75.0	11	84.6	
	c	1	1.8	1	4.2	0	0.0	
Lips spread	a	11	20.0	5	20.8	1	7.7	0.58
	b	30	54.5	12	50.0	9	69.2	
	c	11	20.0	6	25.0	1	7.7	
	d	3	5.5	1	4.2	2	15.4	
Lips sealed	a	27	49.1	11	45.8	5	38.5	0.75
	d	14	25.5	6	25.0	5	38.5	
	c	11	20.0	7	29.2	2	15.4	
	d	3	5.5	0	0.0	1	7.7	
Lips alternate	a	19	34.5	8	33.3	2	15.4	0.82
	b	30	54.5	14	58.3	10	76.9	
	c	5	9.1	2	8.3	1	7.7	
	d	1	1.8	0	0.0	0	0.0	
Lips in speech	a	21	38.2	5	20.8	2	15.4	0.55
	b	30	54.5	17	70.8	10	76.9	
	c	3	5.5	2	8.3	1	7.7	
	d	1	1.8	0	0.0	0	0.0	
Jaw at rest	a	45	81.8	18	75.0	10	76.9	0.76
	b	10	18.2	6	25.0	3	23.1	
Jaw in speech	a	22	40.0	14	58.3	7	53.8	0.57
	b	29	52.7	8	33.3	5	38.5	
	c	4	7.3	2	8.3	1	7.7	
Palate while eating	a	43	78.2	14	58.3	5	38.5	0.017^a^
	b	10	18.2	10	41.7	8	61.5	
	c	2	3.6	0	0.0	0	0.0	
Palate maintenance	a	32	58.2	12	50.0	4	30.8	0.32
	b	22	40.0	10	41.7	8	61.5	
	c	1	1.8	2	8.3	1	7.7	
Palate in speech	a	32	58.2	12	50.0	3	23.1	0.16
	b	21	38.2	11	45.8	10	76.9	
	c	2	3.6	1	4.2	0	0.0	

^a^-statistically significant

**Table 5:** The link between FDA sections and level of education with the use of χ^2^ test. Data are presented as number of cases

FDA indice	Rating	Level of education								*p*
		University (*n* = 17)	%	Secondary (*n* = 28)	%	Trade (*n* = 30)	%	Primary (n = 17)	%	
Cough	a	16	94.1	24	85.7	25	83.3	14	82.4	0.82
	b	1	5.9	3	10.7	4	13.3	2	11.8	
	c	0	0.0	1	3.6	0	0.0	1	5.9	
	d	0	0.0	0	0.0	1	3.3	0	0.0	
Swallowing	a	15	88.2	15	53.6	20	66.7	13	76.5	0.07
	b	1	5.9	13	46.4	8	26.7	4	23.5	
	c	1	5.9	0	0.0	2	6.7	0	0.0	
Drooling	a	8	47.1	7	25.0	11	36.7	5	29.4	0.64
	b	8	47.1	20	71.4	17	56.7	12	70.6	
	c	1	5.9	1	3.6	2	6.7	0	0.0	
Respiration at rest	a	16	94.1	22	78.6	22	73.3	12	70.6	0.49
	b	1	5.9	6	21.4	7	23.3	5	29.4	
	c	0	0.0	0	0.0	1	3.3	0	0.0	
Respiration in speech	a	14	82.4	13	46.4	15	50.0	10	58.8	0.17
	b	2	11.8	13	46.4	13	43.3	4	23.5	
	c	1	5.9	2	7.1	1	3.3	3	17.6	
	d	0	0.0	0	0.0	1	3.3	0	0.0	
Lips at rest	a	13	76.5	5	17.9	9	30.0	2	11.8	0.0002*
	b	4	23.5	23	82.1	19	63.3	15	88.2	
	c	0	0.0	0	0.0	2	6.7	0	0.0	
Lips spread	a	6	35.3	4	14.3	7	23.3	0	0.0	0.16
	b	8	47.1	18	64.3	14	46.7	11	64.7	
	c	2	11.8	5	17.9	8	26.7	3	17.6	
	d	1	5.9	1	3.6	1	3.3	3	17.6	
Lips sealed	a	12	70.6	12	42.9	12	40.0	7	41.2	0.49
	d	3	17.6	8	28.6	10	33.3	4	23.5	
	c	1	5.9	7	25.0	6	20.0	6	35.3	
	d	1	5.9	1	3.6	2	6.7	0	0.0	
Lips alternate	a	11	64.7	6	21.4	11	36.7	1	5.9	0.02*
	b	5	29.4	19	67.9	15	50.0	15	88.2	
	c	1	5.9	3	10.7	3	10.0	1	5.9	
	d	0	0.0	0	0.0	1	3.3	0	0.0	
Lips in speech	a	11	64.7	5	17.9	11	36.7	1	5.9	0.01*
	b	5	29.4	22	78.6	15	50.0	15	88.2	
	c	1	5.9	1	3.6	3	10.0	1	5.9	
	d	0	0.0	0	0.0	1	3.3	0	0.0	
Jaw at rest	a	15	88.2	23	82.1	24	80.0	11	64.7	0.36
	b	2	11.8	5	17.9	6	20.0	6	35.3	
Jaw in speech	a	7	41.2	11	39.3	20	66.7	5	29.4	0.03*
	b	10	58.8	12	42.9	9	30.0	11	64.7	
	c	0	0.0	5	17.9	1	3.3	1	5.9	
Palate while eating	a	15	88.2	18	64.3	16	53.3	13	76.5	0.12
	b	1	5.9	10	35.7	13	43.3	4	23.5	
	c	1	5.9	0	0.0	1	3.3	0	0.0	
Palate maintenance	a	12	70.6	14	50.0	13	43.3	9	52.9	0.09
	b	5	29.4	14	50.0	13	43.3	8	47.1	
	c	0	0.0	0	0.0	4	13.3	0	0.0	
Palate in speech	a	12	70.6	14	50.0	12	40.0	9	52.9	0.30
	b	4	23.5	14	50.0	16	53.3	8	47.1	
	c	1	5.9	0	0.0	2	6.7	0	0.0	

*-statistically significant

## Discussion

Speech disorders in PD tend to vary in character and often have an early onset. Due to such a considerable variety and individual pattern of the disorders it seems advisable to consider all factors contributing to their presence [28]. Assessment of the relationship between age, educational background, sex and place of residence appears to be an important element determining further treatment of PD patients, particularly the non-pharmacological one.

Documented results of research analysing the association between articulation efficiency in PD patients and their age are scarce and controversial [8, 9, 28]. A group of elderly healthy subjects were compared to a group of younger PD patients. The former were found to have a lower mobility of the jaw and lips than their younger counterparts [29, 30]. Other studies, however, did not confirm the findings [8]. Also, an analysis of similar articulation regions in PD patients of various ages showed that the older patients have lower mobility within the jaw, which consequently affects the quality of respiratory and phonation functions [11].

Our study however revealed no correlation between age and the performance of the speech organs.

We carried out the research in an attempt to establish the impact of educational background on the efficiency of speech organs. No relevant scientific publications are available in existing literature. We found that highly educated patients tended to display increased efficiency in proper positioning of the lips at rest, a lower severity of disorders affecting the movements of the vocal organs and lower jaw during the production of speech. This might be due to a higher communicative awareness prevailing in the group, which results in more care being taken to proper movement of the speech organs. The likelihood of PD patients controlling their speech has been mentioned by other authors [31]. This feature probably contributes to an increased ability to correct articulation inaccuracies among highly educated patients.

It has been scientifically proven that the course of PD in males is not the same as in females. Recent research has revealed that men are more likely than women to lose their voice loudness and the quality of speech. They also take more pauses while speaking [32] and display disturbed prosody [33, 34]. Evaluation of the efficiency of the larynx in men and women showed that women display a higher incidence of phonation disorders, glottis insufficiency and frequent laryngeal tremor. The differences might be attributed to a possibly faster progression of the disease in men as well as an increased number of abnormalities within the articulatory organs.

Acoustic voice analysis in PD patients confirmed the difference between the fundamental frequency in men and women [35]. The study revealed that there is no difference between the performance of the articulatory organs in men and women [36–43].

We were also first to try to establish the existence of a link between the efficiency of the speech organs in PD patients and their place of residence. Our study revealed that city dwellers displayed significantly better performance of the soft palate while eating, which probably results from the fact that those patients tend to take a better care of proper verbal expression, articulation, or are better educated. It could also be that they have better access to medical treatment and medication control, just because healthcare is better in urban areas.

It may be also connected with a higher incidence of PD in rural areas. The use of pesticides and presence of heavy metals in well water are often quoted as contributory environmental factors of PD incidence in human population. Since well water is hardly ever used for consumption in Poland, pesticides and heavy metals might account for the main threat. Exposure to environmental risk factors might accelerate the onset of PD disorders, which in turn results in a higher prevalence of impaired mobility of the articulatory organs [44–46].

A higher number of abnormalities in the functioning of the articulatory organs occurring among the residents of rural areas might result from a poorer access to healthcare, and consequently delays in starting proper treatment [47–49].

Our study of the impact of environmental factors on the severity of the articulatory organs disorders in PD individuals is by no means exhaustive. It is just an introduction to further research based on more precise and accurate methodology. However, detecting the small changes in the articulatory organs might contribute to an early diagnosis of PD.

## Conclusions

The study revealed significant association between the functionality of articulators in PD patients and their educational background and place of residence. Big city dwellers demonstrated lower incidence of disorders within speech organs, particularly those affecting mobility of the soft palate while eating. Disorders of moderate intensity were more frequently found in subjects living in villages. Subjects with university education displayed better position of the lips at rest and better performance of both the lips and the mandible while speaking.

## Abbreviations

CT: Computed tomographic
DBS: Deep brain stimulation procedure
FDA: Frenchay Dysarthria Assessment
H&Y: Scale M.M. Hoehn and M.D. Yahr
MDS-UPDRS: Movement Disorder Society -Unified Parkinson’s disease rating scale
MRI: Magnetic resonance imaging
PD: Parkinson’s disease
UKPDSBB: The United Kingdom Parkinson’s Disease Society Brain Bank
